# Fructose consumption and metabolic hypertension in rats: a systematic review and meta-analysis

**DOI:** 10.7717/peerj.20097

**Published:** 2025-12-19

**Authors:** Arya VS, Arunkumar Subramanian, Kavitha Rajendran, Tamilanban Thamaraikani, Vinoth Kumarasamy, Mok Shiueh Lian, Rusli Bin Nordin, Mahendran Sekar, Vetriselvan Subramaniyan, Manasa Karnam, Steni Sackiriyas, Ling Shing Wong

**Affiliations:** 1 Department of Pharmacology, SRM College of Pharmacy, SRM Institute of Science and Technology, Chengalpattu, Tamil Nadu, India; 2 Department of Pharmaceutics, SRM College of Pharmacy, SRM Institute of Science and Technology, Chengalpattu, Tamil Nadu, India; 3 Department of Pharmacology, Faculty of Medicine, MAHSA University, Bandar Saujana Putra, Selangor, Malaysia; 4 Department of Parasitology & Medical Entomology, Faculty of Medicine, Universiti Kebangsaan Malaysia, Jalan Yaacob Latif, Kuala Lumpur, Malaysia; 5 School of Pharmacy, Monash University, Bandar Sunway, Subang Jaya, Selangor, Malaysia; 6 Department of Medical Sciences, School of Medical and Life Sciences, Sunway University, Bandar Sunway, Selangor, Malaysia; 7 Department of Pharmacology, MNR College of Pharmacy, Sangareddy, Telangana, India; 8 Physical Therapy Program, Health Science Center, University of Wisconsin-La Crosse, WI, United States of America; 9 Faculty of Health and Life Sciences, INTI International University, Nilai, Malaysia

**Keywords:** Metabolic hypertension, High fructose diet, Cardiovascular disorders, Blood pressure, Public health

## Abstract

**Background:**

Hypertension is a growing cardiovascular risk, increasingly linked to dietary changes. This meta-analysis examines the impact of dietary fructose on hypertension by evaluating the mean difference in systolic blood pressure (SBP) in rats, considering fructose intake levels (10–30% in solution; 60–75% in diet) and exposure duration (<8 weeks, 8 weeks and >8 weeks).

**Materials and Methods:**

A comprehensive search across Cochrane, Web of Science, Scopus and Ovid (MEDLINE, Embase, AMED) databases identified relevant reports published until December 2022. The meta-analysis, conducted using Review Manager 5.4 software, included a total of 24 studies, with quantitative data analysed through a random-effects model.

**Results:**

Fructose treatment significantly raised SBP in rats by 31.05 mmHg (95% CI [24.36–37.74], *P* < 0.00001), with an *I*^2^ value of 100%, indicating high heterogeneity. Subgroup analysis showed SBP increases of 28.50 mmHg (95% CI [15.25–41.75]) for 10–30% w/v fructose solution and 33.80 mmHg (95% CI [28.27–39.33]) for 60–75% w/v fructose diet, reinforcing a strong link to hypertension and suggesting a dose-dependent effect. Additionally, analysis based on fructose administration duration confirmed a significant SBP increase, underscoring its impact.

**Conclusion:**

Animal studies suggest that fructose intake may contribute to elevated SBP, potentially increasing the risk of hypertension. While trends were observed across various doses and exposure durations, not all findings reached statistical significance. These observations highlight the importance of further research into the long-term effects of fructose on human blood pressure and their relevance to dietary guidelines and public health policies.

## Introduction

Metabolic disorders such as hypertension, hyperglycemia, dyslipidemia, and hypertriglyceridemia are increasingly linked to excessive fructose consumption (Johnson et al., 2007; Taskinen et al., 2017). Unlike glucose, fructose undergoes distinct metabolic processing, which has raised concerns about its role in disease pathogenesis (Malik & Hu, 2015). Fructose, a monosaccharide found naturally in fruits, is also a key component of table sugar and high-fructose corn syrup (HFCS). However, it is important to distinguish between the fructose found in whole fruits and that present in table sugar or HFCS. While chemically similar, the fructose in fruits is accompanied by dietary fibre, antioxidants, vitamins, and minerals, which can attenuate its absorption and mitigate adverse metabolic effects. In contrast, the fructose from added sugars (like sucrose or HFCS) is absorbed rapidly and has been more strongly associated with metabolic disorders. Thus, the health impacts of fructose may vary depending on its dietary source (Madsen, Sen & Aune, 2023).

The widespread adoption of Western dietary patterns has led to a surge in fructose intake, primarily through processed foods and sugar-sweetened beverages (Marriott, Cole & Lee, 2009). This shift has coincided with an increasing prevalence of metabolic syndrome, particularly in regions like India (Gulati & Misra, 2014). Recent studies are indicating that the addition of fructose in large amounts to a regular diet may end up with undesirable health outcomes (Bantle, 2009).

High blood pressure or hypertension plays a crucial role in metabolic syndrome and significantly elevates the risk of early morbidity and mortality from stroke, cardiovascular diseases, and kidney disorders (Cornier et al., 2008; Saklayen, 2018). According to the National Health and Nutrition Examination Survey (NHANES 2003–2006), excessive fructose consumption from added sugars is linked to higher blood pressure levels in people who have never had hypertension (Jalal et al., 2010). According to published reports, increased fructose consumption plays a key role in the development of hypertension in metabolic syndrome in both rodents and humans (Vasdev et al., 1994; Palumbo et al., 1977; Hwang et al., 1987; Dai & McNeill, 1995; Catena et al., 2003; Visvanathan et al., 2005; Brown et al., 2008). Large epidemiological studies have shown that dietary fructose from sucrose or HFCS in foods and beverages has been convincingly linked to hypertension (Jalal et al., 2010). Hypertension is a major risk factor for cardiovascular complications (Zanchetti, 2017), so it should be taken care of properly to avoid the development and progression of disease.

**Figure 1:**
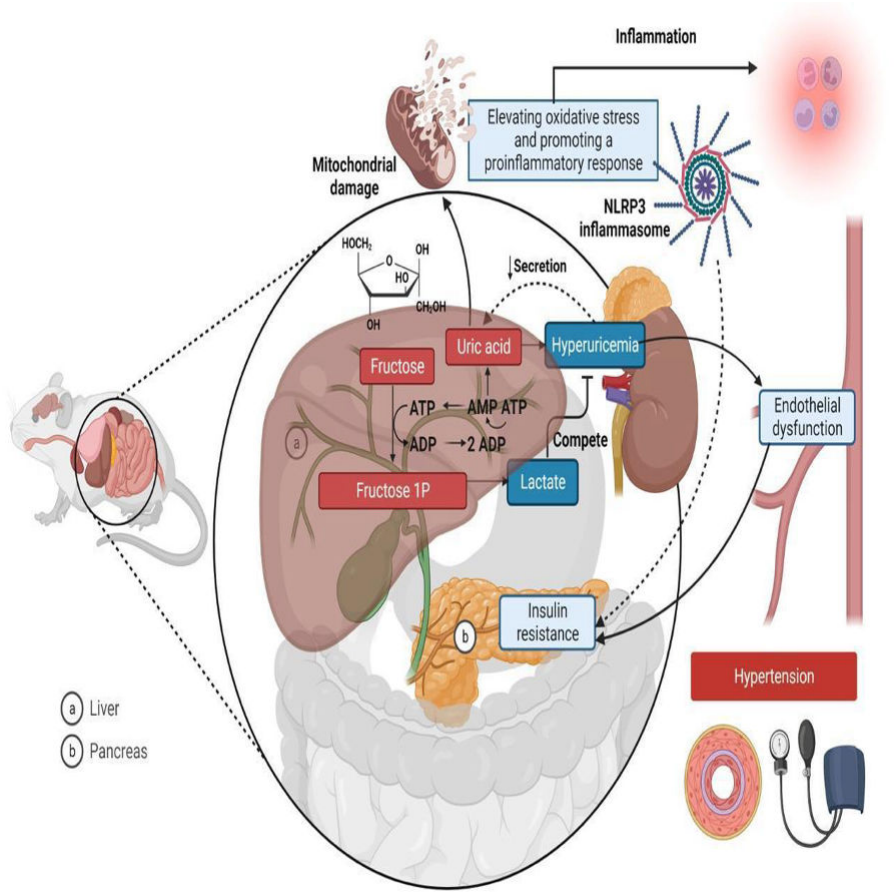
Mechanisms linking fructose metabolism to metabolic hypertension in rats. In the liver (A), fructose is phosphorylated into fructose-1-phosphate (Fructose 1P) using Adenosine Triphosphate (ATP), leading to ATP depletion and increased Adenosine Monophosphate (AMP) degradation, which generates uric acid as a byproduct. Excess uric acid enters circulation, contributing to hyperuricemia, endothelial dysfunction, and hypertension. Additionally, fructose metabolism increases lactate production, which competes with uric acid for renal excretion. Uric acid also induces mitochondrial damage and oxidative stress, activating the NLR family-pyrin domain-containing 3 (NLRP3) inflammasome and promoting a pro-inflammatory response. In the pancreas (B), fructose metabolism contributes to insulin resistance, further exacerbating metabolic dysfunction. Together, these processes link fructose consumption to hypertension, inflammation, and cardiometabolic diseases. Created in Biorender.

Rodent models are widely used in metabolic syndrome studies (Panchal & Brown, 2011; Wong et al., 2016) because they exhibit physiological and metabolic characteristics similar to humans, enabling the study of disease mechanisms and potential treatments in a controlled setting (Leibowitz et al., 2013). Fructose is known to cause hypertension in metabolic syndrome through various mechanisms, including elevating oxidative stress, promoting inflammatory responses, inducing hyperuricemia, causing insulin resistance, and ultimately leading to endothelial dysfunction and metabolic hypertension (Fig. 1) (Klein & Kiat, 2015; Soleimani et al., 2023). In rat studies, systolic blood pressure (SBP) values serve as the basis for classifying hypertension. A measurement of 114 mmHg or lower is regarded as normotensive, while an SBP of 115 mmHg or above indicates hypertension (Kapsdorferová, Grešová & Švorc, 2024; Tukhovskaya et al., 2024).

Despite ongoing research, uncertainties persist regarding the exact mechanisms by which fructose contributes to hypertension (Fattore et al., 2017). Many studies in rats have used varying concentrations of fructose, administered through different methods, such as 60–75% w/v as a chow component or 10–30% w/v as a fructose beverage, to induce metabolic syndrome (Abdulla, Sattar & Johns, 2011).

To gain deeper insights into how fructose intake influences key cardiovascular risk factors, particularly hypertension, we conducted a systematic review and meta-analysis. This study examines the relationship between fructose consumption and increases in SBP by evaluating different doses—10–30% w/v as a solution and 60–75% w/v as part of the diet—alongside varied durations of administration (<8 weeks, 8 weeks, and >8 weeks). While Toop & Gentili (2016) examined the impact of fructose consumption duration on blood pressure changes across slightly different time frames, our study provides further insights into how fructose intake at both low (10–30% w/v solution) and high (60–75% w/v diet) doses affects blood pressure. In contrast to their research, our investigation specifically evaluates the dose-dependent effect of fructose on SBP, offering a more focused assessment of hypertension risk (Toop & Gentili, 2016).

Based on existing evidence, we hypothesize that fructose consumption increases systolic blood pressure in rats, with the magnitude of this effect influenced by both the dose and duration of fructose administration. This meta-analysis systematically evaluates this relationship to provide quantitative insights into its impact on hypertension risk.

## Materials and Methods

**Figure 2:**
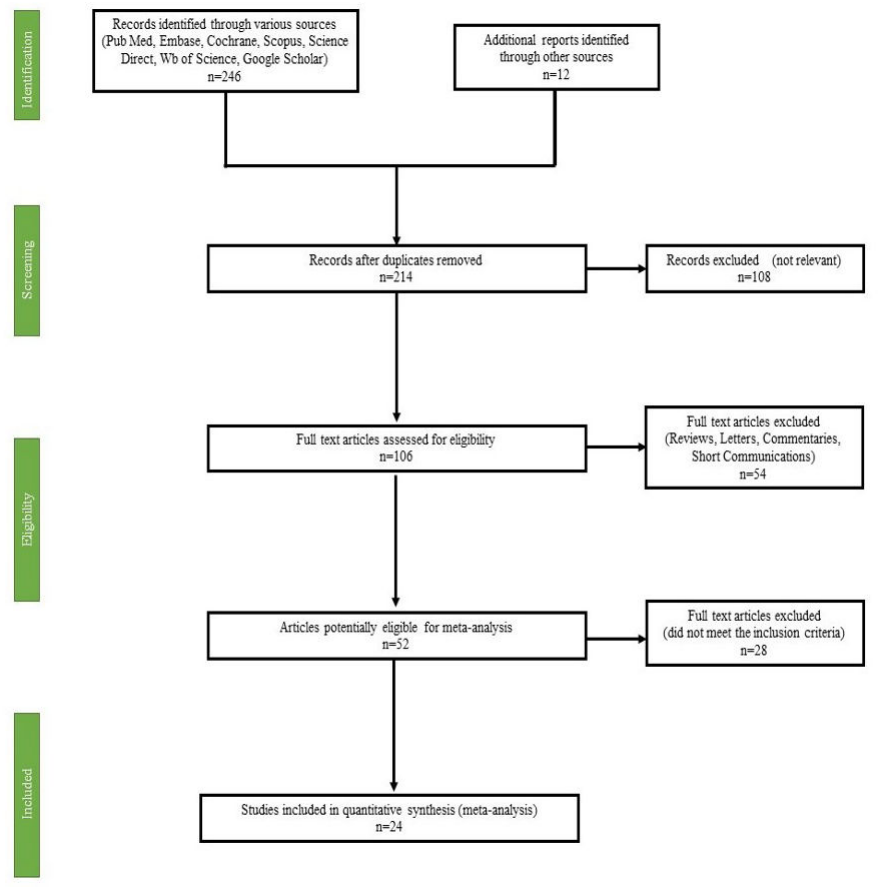
PRISMA flow diagram of study selection for systematic review and meta-analysis.

This systematic review and meta-analysis adhered to the guidelines set forth by the Preferred Reporting Items for Systematic Reviews and Meta-Analyses (PRISMA). The PRISMA flowchart (Fig. 2) illustrates this selection process in detail.

### Search strategy and selection of studies

This systematic review and meta-analysis aim to evaluate the association between fructose consumption and the development of hypertension in animals based on previous research findings. To achieve this, data were gathered from various international databases, including Scopus, Embase, PubMed/MEDLINE, Science Direct, Web of Science, Cochrane Library, and Google Scholar, covering studies published between 2010 and 2022.

To ensure a comprehensive search, relevant keywords were used, such as “Fructose”, “Hypertension”, “Endothelial dysfunction”, “Rats”, “Fructose feeding”, “Fructose consumption”, “Metabolic Syndrome”, and “Fructose and Systolic Blood Pressure (SBP)”. A combination of Boolean operators “AND” and “OR” facilitated an inclusive search strategy.

From the initial search, 258 articles were identified. After the removal of 44 duplicate studies, the remaining 214 articles underwent screening based on predetermined eligibility criteria. Following this screening process, 24 studies met the inclusion criteria and were included in the final meta-analysis (Fig. 2). The selection criteria for studies included those involving rats of either Wistar or Sprague Dawley strains, regardless of sex. Studies had to investigate fructose administration, either as a solution (10–30%) or in the diet (60–75%), over a duration of 2–14 weeks. To ensure meaningful comparisons, studies were required to include a normal control group alongside the fructose-fed group, with both groups receiving treatment consistently throughout the study duration. Furthermore, only studies presenting data in tabulated form or graphical figures, including standard deviation (SD) or standard error mean (SEM), were considered. This review follows a structured protocol, which has been registered on PROSPERO under the registration number CRD42024437704, ensuring methodological transparency and rigor in the research process.

### Inclusion and exclusion criteria

Inclusion criteria were fixed based on the Population, Intervention, Comparison, and Outcome (Richardson et al., 1995; Reshma et al., 2024) where (i) Population: Rats (either normotensive at baseline or those exposed to fructose to assess hypertension development) (ii) Intervention: Administration of fructose (as a solution or diet) at varying concentrations and durations (iii) Comparison: Control group receiving a normal diet without fructose exposure and (iv) Outcome: Change in SBP following fructose administration, expressed as a continuous outcome (standardized mean difference).

The exclusion criteria were: (i) Fructose feeding in combination with other sweeteners or fat. (ii) Fructose consumption along with other chemicals, (iii) Short communications, case reports, mini and full-length reviews, comments and letter to the editors and (iv) Articles without full texts or in any other languages other than English. (v) Articles with number of animals per group less than 4.

### Data collection

Two reviewers autonomously evaluated the titles and/or abstracts of publications acquired through the search methodology. Two writers from the review team individually retrieved and thoroughly evaluated the complete text of these potentially eligible studies in order to ascertain their eligibility. Any discrepancy between the two reviewers on the suitability of particular research were resolved through deliberation and agreement. If a resolution was not reached, an additional reviewer was included to make a final decision. The mean and standard deviation values for SBP in control and fructose-fed animals were extracted using Graph Reader (http://www.graphreader.com/) and Plot Digitizer (https://plotdigitizer.com/extrac-data-graph-image). These tools allowed precise data retrieval from graphical representations. The key effects of fructose on hypertension are summarized in Table 1.

**Table 1:** Summary of 24 studies included in the systematic review and meta-analysis on fructose administration and hypertension.

**Author Name & Year**	**No. of animals per treatment group (n)**	**Species of rat**	**Concentration of fructose**	**Duration of administration**	**SBP of control animal (mmHg)**	**SBP of the fructose treated animal (mmHg)**	**Experimental condition**
Jensen et al. (2022)	5	Sprague-Dawley (SD)	10%	14 weeks	118 ± 5	126 ± 7	• Male SD rats of 4 weeks age were used • Control animal received no treatment or vehicle • Treatment group received 10% w/v fructose as solution
Kho et al. (2014)	10	SD	65%	8 weeks	96 ± 1.21	136.71 ± 1.24	• Seven-week-old male Sprague-Dawley (SD) rats were used • Control group-no treatment • Treatment group received 65% fructose as diet for 8 weeks
Chou et al. (2013)	6	Wistar–Kyoto	65%	8 weeks	108 ± 3	141 ± 5	• Male Wistar–Kyoto rats of 200–230 g, were used for the experiments. • Group Control: rats were fed standard chow diet for 8 weeks • Group Fructose: rats were fed the 65% fructose diet for 8 weeks
Kho et al. (2016a)	10	SD	60%	8 weeks	108 ± 1.1	135.7 ± 1.3	• Seven-week-old male Sprague–Dawley (SD) rats were used • Group Control: rats were fed standard chow diet for 8 weeks • Group Fructose: rats were fed the 60% fructose diet for 8 weeks
Kho et al. (2016b)	10	SD	60%	8 weeks	115 ± 0.2	145 ± 0.9	• Seven-week-old male Sprague–Dawley (SD) rats were used • Group Control: rats were fed standard chow diet for 8 weeks • Group Fructose: rats were fed the 60% fructose diet for 8 weeks
Dos Santos et al. (2018)	10	Wistar	10%	12 weeks	120.80 ± 1.07	143.05 ± 3.21*	• Male Wistar rats weighing around 150 g were used • Control-no treatment • Treatment −10% fructose for 12 weeks
Dupas et al. (2017)	6	Wistar	20%	4 weeks	122.7 ± 2.2	136.3 ± 1.8	• Male Wistar rats: 3 weeks old were used • Control: no treatment • Treatment: 20% w/v solution of fructose for 4 weeks
Palanisamy & Venkataraman (2013)	6	Wistar	60%	8 weeks	115.6 ± 8.9	189.2 ± 12.5	• Adult male Wistar rats of body weight 150–160g were used • Control diet containing 60% maize starch, 20% protein, 0⋅7% methionine, 5% ground nut oil, 10⋅6% wheat bran, 3⋅5% salt mixture and 0⋅2% vitamin mixture and given the vehicle • Treatment group received 60% fructose instead of starch in the control diet
Mahmoud & Elshazly (2014)	8	Wistar	10%	12 weeks	105 ± 2.4	140 ± 5.9	• Adult male Wistar rats (140–160 g) • Control: rats received vehicle only • Treatment: rats received the vehicle and administered fructose 10% in drinking water for 12 weeks
Chou et al. (2015)	6	Wistar	60%	8 weeks	118 ± 4.7	148 ± 4.6	• Male Wistar-Kyoto rats • Con: standard chow diet for 8 weeks • Treatment: 60% fructose diet for 8 weeks
Yeh et al. (2014)	6	Wistar	10%	4 weeks	126.6 ± 2.8	155.7 ± 4.9	• Wistar-Kyoto (WKY) rats at 6 weeks of age • Fructose group consisting 14 of WKY rats fed 10% fructose in drinking water for 4 weeks • Control: no treatment
Rehakova et al. (2016)	6	Wistar-Kyoto	15%	6 weeks	119.6 ± 1.33	124.3 ± 2.79	• Male 12-week-old Wistar rats • Control: tap water for 6 weeks • Treatment: 15% fructose solution for 6 weeks
Ho et al. (2020)	8	Wistar-Kyoto	10%	4 weeks	109.8 ± 1.8	144.5 ± 1.1	• Wistar rats were used • Control: regular tap water • Treatment: 10% fructose solution for 2–4 weeks
Mondal, Gowda & Manandhar (2019)	6	Wistar	10%	6 weeks	111.1 ± 8.229	174.1 ± 8.079	• Male Albino Wistar rats weighing about 110–150 g were used • Control: tap water for 6 weeks • Treatment: 10% fructose for 6 weeks
Bhagya, Prema & Rajamohan (2012)	6	SD	71%	3 weeks	119 ± 2	145 ± 2	• Male Sprague- Dawley rats weighing between 150–170 g were used for the study • Control diet: 71% corn starch, 8% fat and 16% protein • Treatment: 71% fructose, 8% fat and 16% protein.
Yoro et al. (2020)	6	Wistar	60%	4 weeks	103.3 ± 1.05	139.3 ± 6.15	• Wistar strain, aged from 8–12 weeks and weighing on average between 140 and 180 g were used • Control: tap water for 30 days • Treatment: 60% solution of fructose for 30 days
Zhang et al. (2021)	4	SD	60%	14 weeks	93.5 ± 5.8	131.8 ± 5.5	• Male Sprague-Dawley (SD) rats (6–8 wks old, 150–200 g) • Control: no treatment • Treatment: high fructose diet
Zemančíková & Török (2014)	8	Wistar	10%	8 weeks	118.1 ± 2.0	124.6 ± 1.6	• Twelve-week-old male Wistar rats were used • Control: tap water for 8 weeks • Treatment: 10% fructose solution for 8 weeks
Virupaksha (2017)	6	Wistar	10%	3 weeks	120.6 ± 0.81	187 ± 0.89	• Wister albino rats of either sex, weighing around 200–250 g. were used in the study • Control: tap water for 3 weeks • Treatment: 10% fructose solution for 21 days
Ghibu et al. (2013)	10	SD	60%	12 weeks	113.66 ± 1.14	130.61 ± 0.80	• Male Sprague-Dawley rats (7 weeks old), weighing 200–250 g was used • Control group received regular chow • High fructose-fed group received chow supplemented with fructose 60%
Bertera et al. (2012)	6	SD	10%	6 weeks	105 ± 2	114 ± 2	• Male Sprague–Dawley rats were used (220–250 g) • Control with tap water to drink for 6 weeks • Fructose: treated with fructose solution (10% w/v) to drink for 6 weeks
Mamikutty et al. (2014)	6	Wistar	20%	8 weeks	103.3 ± 1.1	145.8 ± 1.5	• Male Wistar rats with a body weight between 250 and 300 g were used • Control with tap water to drink for 8 weeks • Fructose: treated with fructose solution (20% w/v) to drink for 8 weeks
Chou et al. (2017)	6	Wistar	10%	8 weeks	110 ± 3	141 ± 4	• Male Wistar ± Kyoto rats with the initial weight of 200 ± 230 g, were used • Control: tap water for 8 weeks • Treatment: 10% fructose solution for 8 weeks
Kanthlal et al. (2021)	6	SD	10%	8 weeks	123 ± 8	157 ± 6	• Male SD rats were used • Control: tap water for 8 weeks • Treatment: 10% fructose solution for 8 weeks • Data of SBP extracted digitally from graphs

### Quality assessment evidence

The studies considered in this analysis had several common shortcomings, including poor randomization, inadequate allocation concealment, absence of caregivers/investigators blinding, and inadequate reporting of baseline variables. The information regarding the housing or caging of experimental animals is insufficient. The SYRCLE’s RoB tool, created by the Systematic Review Centre for Laboratory Animal Experimentation, was employed to assess the risk of bias in the included animal studies, we used SYRCLE’s Risk of Bias (RoB) tool, which is specifically designed for preclinical studies. This tool evaluates key domains, including: selection bias, performance bias, detection bias, attrition bias, and reporting bias (Hooijmans et al., 2014).

### Statistical analysis

This study aimed to evaluate changes in systolic blood pressure in rats before and after fructose consumption. Data analysis was conducted using Review Manager (version 5.4, Cochrane Collaboration), with statistical significance set at *P* < 0.05. The meta-analysis expressed effect size as the mean difference with a 95% confidence interval (CI), calculated by pooling the mean change from baseline and corresponding standard deviations (SDs). To assess persistence and continuous outcomes, the random effects model (REM) was applied. Heterogeneity in effect sizes was primarily examined using the I^2^ statistic and Tau^2^ (Higgins, 2003). Subgroup analyses were performed based on fructose dosage and varying administration durations to explore potential contributors to heterogeneity. Additionally, publication bias was evaluated through visual assessment using a funnel plot.

## Results

### Characteristics of the studies

Figure 2 depicts the screening and review process. Initially, titles and abstracts were examined, leading to the exclusion of 44 irrelevant articles. The remaining 214 article underwent a rigorous evaluation by at least two authors, resulting in the removal of 190 studies due to missing data, inappropriate article types, non-relevant populations, incorrect study durations, intervention-based research designs, or unavailability of the full text. Ultimately, 24 studies were selected for the final systematic review.

All 24 studies utilized fructose as an inducing agent for metabolic syndrome or hypertension, aiming to evaluate the effects of new drug moieties on these conditions. Among them, more than half employed a 10–30% w/v fructose solution, while 42% incorporated fructose at concentrations between 60–75% w/v in the diet to induce hypertension or metabolic syndrome in rats. Regarding study duration, nine studies were conducted for fewer than 8 weeks, 10 lasted exactly 8 weeks, and five extended beyond 8 weeks, all focusing on fructose-induced hypertension and metabolic syndrome. Table 1 provides an overview of the characteristics of the selected articles.

### Risk of bias assessment

**Figure 3:**
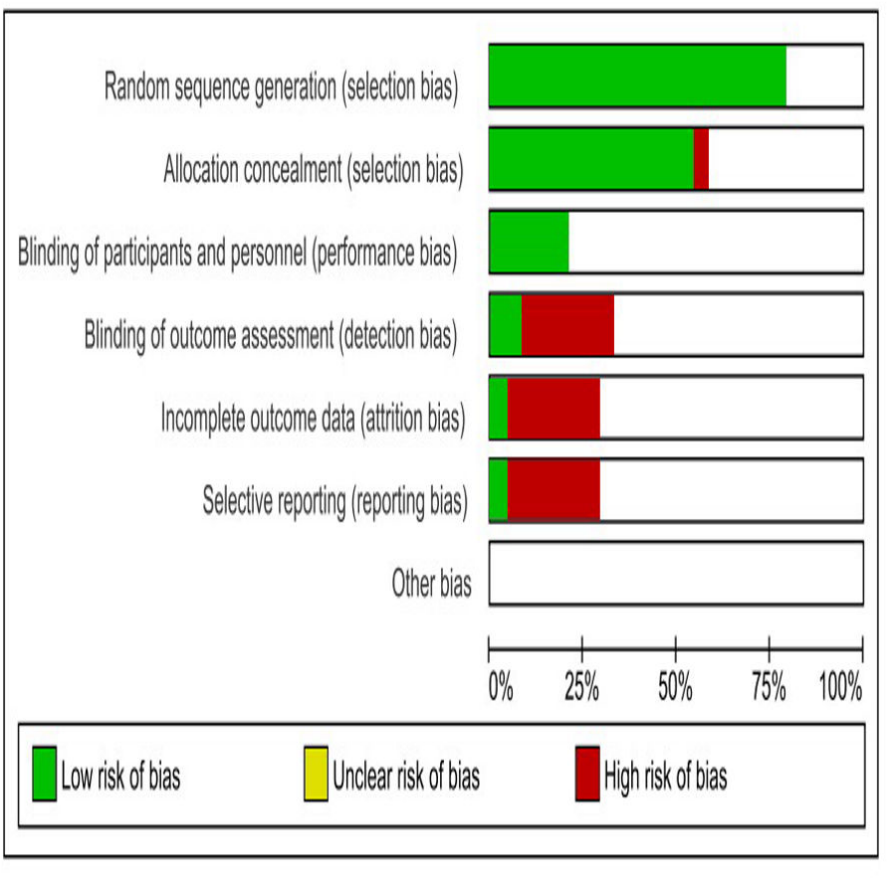
SYRCLES risk of bias assessment for included studies. The horizontal bars indicate the proportion of studies categorized as having a low risk of bias (green), unclear risk of bias (yellow), and high risk of bias (red) for each domain.

Figure 3 illustrates the primary sources of bias identified across the included studies, notably the absence of blinding in outcome assessment, incomplete outcome data, and selective reporting.

### Quantitative data analysis

**Figure 4:**
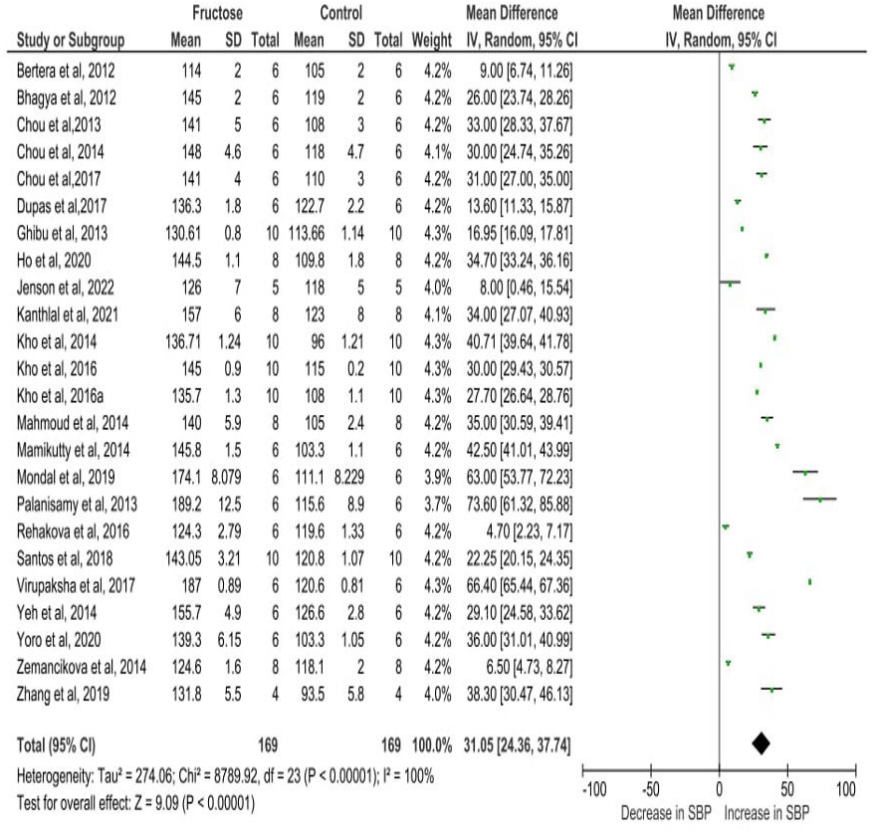
Cumulative effect of fructose on SBP with 95% CI according to the random effect model. CI, Confidence Interval; MD, Mean Difference. Note. Bertera et al., 2012; Bhagya, Prema & Rajamohan, 2012; Chou et al., 2013; Chou et al., 2015; Chou et al., 2017; Dupas et al., 2017; Ghibu et al., 2013; Ho et al., 2020; Jensen et al., 2022; Kanthlal et al., 2021; Kho et al., 2014; Kho et al., 2016a; Kho et al., 2016b; Mahmoud & Elshazly, 2014; Mamikutty et al., 2014; Mondal, Gowda & Manandhar, 2019; Palanisamy & Venkataraman, 2013; Rehakova et al., 2016; Dos Santos et al., 2018; Virupaksha, 2017; Yeh et al., 2014; Yoro et al., 2020; Zemančíková & Török, 2014; Zhang et al., 2021.

Figure 4 depicts the meta-analysis findings, utilizing a random effects model to evaluate the impact of fructose on the SBP of rats. Fructose treatment led to a substantial rise in SBP, with an observed increase of 31.05 mmHg (95% CI [24.36–37.74]). The statistical significance of this effect was highly robust (*P* < 0.00001), indicating a strong association between fructose intake and elevated SBP. Moreover, the analysis revealed considerable heterogeneity among the included studies, with an I^2^ value of 100%, suggesting substantial variations in effect sizes across different experimental conditions. This high heterogeneity underscores the potential influence of diverse factors such as variations in study design, dosage, duration of fructose administration, and animals used in the analysis.

### Sub-category analysis

**Figure 5:**
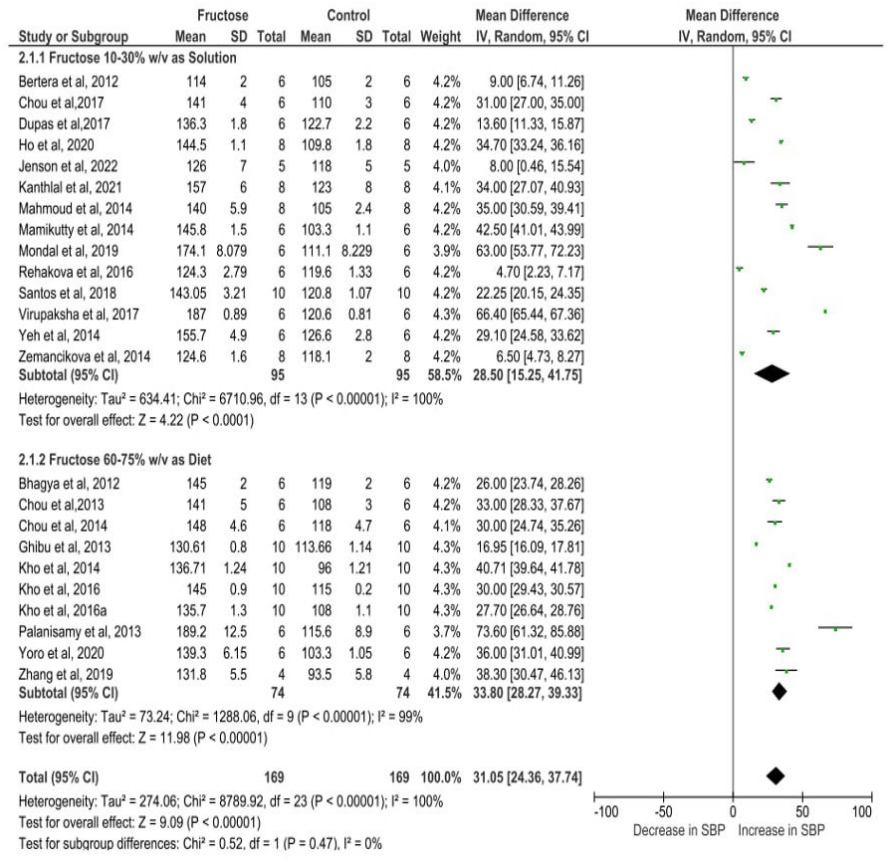
The subgroup analysis of different dose of fructose on SBP. The pooled effects estimates are represented by three diamonds; one for studies with solution, one for studies with diet, and one representing the combined effect. Data are represented as MD with 95% confidence interval. Note. Bertera et al., 2012; Bhagya, Prema & Rajamohan, 2012; Chou et al., 2013; Chou et al., 2015; Chou et al., 2017; Dupas et al., 2017; Ghibu et al., 2013; Ho et al., 2020; Jensen et al., 2022; Kanthlal et al., 2021; Kho et al., 2014; Kho et al., 2016a; Kho et al., 2016b; Mahmoud & Elshazly, 2014; Mamikutty et al., 2014; Mondal, Gowda & Manandhar, 2019; Palanisamy & Venkataraman, 2013; Rehakova et al., 2016; Dos Santos et al., 2018; Virupaksha, 2017; Yeh et al., 2014; Yoro et al., 2020; Zemančíková & Török, 2014; Zhang et al., 2021.

Studies assessing SBP in rats exposed to 10–30% w/v fructose demonstrated a mean difference of 28.50 mmHg (95% CI [15.25–41.75]). In contrast, those receiving a diet containing 60–75% w/v fructose exhibited a slightly higher mean difference of 33.80 mmHg (95% CI [28.27–39.33]). Both groups showed a statistically significant association with SBP elevation, with a *P*-value of less than 0.00001, indicating a strong relationship between fructose intake and increased SBP. The slightly elevated SBP observed with higher fructose doses suggests a potential dose-dependent increase in blood pressure, indicating that greater fructose intake may further amplify the risk. Additionally, the comparison between the two different dose treatment groups was conducted using a test for subgroup differences. The analysis resulted in a *P*-value of 0.47, indicating the absence of a statistically significant difference between the groups. Furthermore, the I^2^ value was 0%, demonstrating no heterogeneity in the subgroup comparisons. These findings suggest that both fructose concentration groups (10–30% and 60–75%) had comparable effects on systolic blood pressure, with no substantial variation in their impact (Fig. 5).

**Figure 6:**
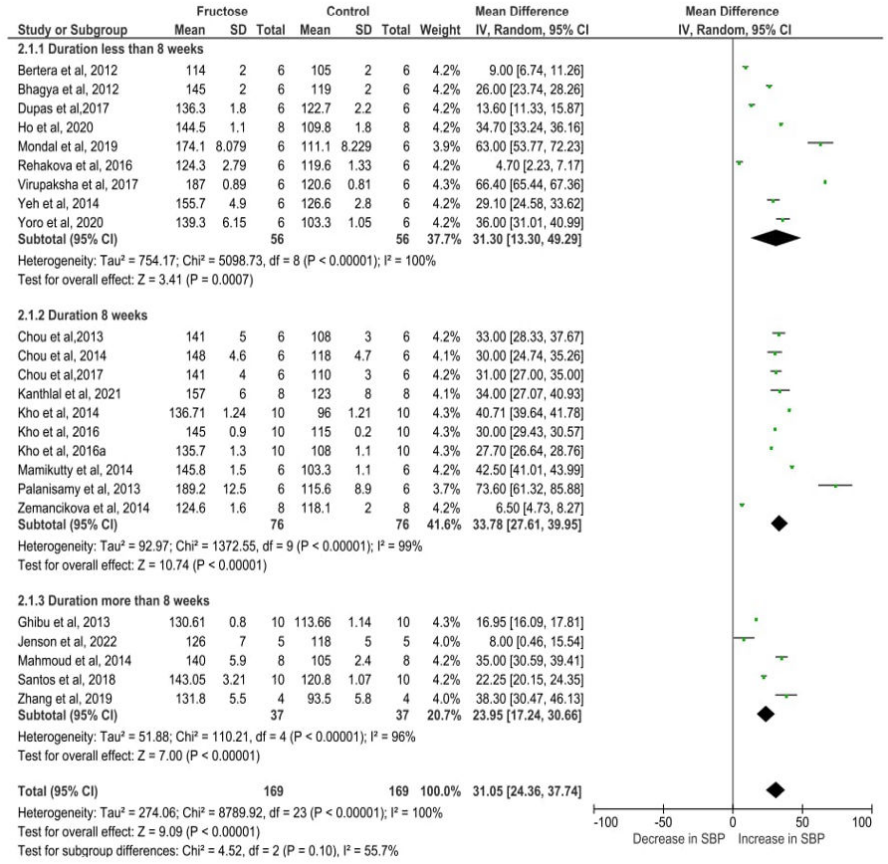
The subgroup analysis on different duration of fructose administration on SBP. The pooled effects estimates are represented by three diamonds; one for studies with less than 8 weeks, one for studies with 8 weeks and one for the studies with more than 8 weeks. One diamond representing the combined effect. Data are represented as MD with 95% confidence interval. Note. Bertera et al., 2012; Bhagya, Prema & Rajamohan, 2012; Chou et al., 2013; Chou et al., 2015; Chou et al., 2017; Dupas et al., 2017; Ghibu et al., 2013; Ho et al., 2020; Jensen et al., 2022; Kanthlal et al., 2021; Kho et al., 2014; Kho et al., 2016a; Kho et al., 2016b; Mahmoud & Elshazly, 2014; Mamikutty et al., 2014; Mondal, Gowda & Manandhar, 2019; Palanisamy & Venkataraman, 2013; Rehakova et al., 2016; Dos Santos et al., 2018; Virupaksha, 2017; Yeh et al., 2014; Yoro et al., 2020; Zemančíková & Török, 2014; Zhang et al., 2021.

The increase in SBP varied depending on the duration of fructose administration in rats. In studies with a treatment period of less than 8 weeks, SBP rose by 31.30 mmHg (95% CI [13.30–49.29], *P* = 0.0007). For studies lasting 8 weeks, the increase was slightly higher at 33.78 mmHg (95% CI [27.61–39.95], *P* < 0.00001). However, in studies extending beyond 8 weeks, the elevation in SBP was comparatively lower at 23.95 mmHg (95% CI [17.24–30.66], *P* < 0.00001). Despite the variation in SBP elevation across different durations, the statistically significant results indicate that fructose consumption consistently leads to increased blood pressure in rats, regardless of the length of exposure (Fig. 6). Additionally, the test for subgroup differences yielded a *P*-value of 0.10 and an I^2^ value of 55.7%, suggesting that there is no significant difference in SBP elevation across the different durations of fructose treatment.

### Publication bias assessment

**Figure 7:**
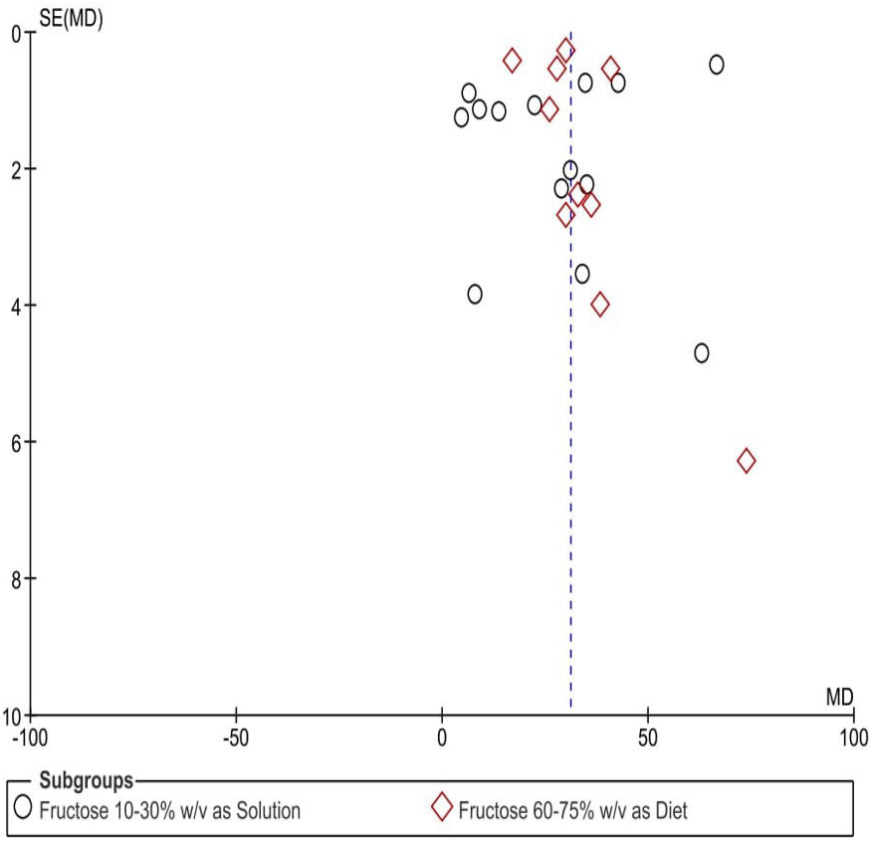
Funnel plots depicting the impact of fructose on hypertension in animal-related studies, selected according to criteria set for this research. Each point in the funnel plots represents an individual study.

A visual evaluation of the funnel plots (Fig. 7) indicated potential publication bias across the assessed parameters. The uneven distribution of data points suggests inconsistencies in study result reporting, raising concerns about selective publication or systematic distortions.

## Discussion

Fructose is often employed in animal studies to replicate characteristics of metabolic syndrome including metabolic hypertension (Sánchez-Lozada et al., 2007; Wong et al., 2016; Taskinen, Packard & Borén, 2019). However, the physiological effects of fructose can differ based on the concentration given and the method of administration. The differences in study design have resulted in inconsistent findings regarding the effects of fructose consumption, making it challenging to extrapolate and comprehend its impact on human health. Our meta-analysis demonstrated that the intake of fructose, regardless of study design variations, concentration, and duration, significantly raises the SBP in rats.

Consuming foods high in fructose has the propensity to raise blood pressure, which raises the risk of major cardiovascular events and mortality because hypertension is the main risk factor for negative outcomes and has a substantial impact on cardiovascular health (Busnatu et al., 2022). Additionally, even mild increases in blood pressure within the normal range can significantly impact cardiovascular health (Perret-Guillaume, Joly & Benetos, 2009; Buckley et al., 2023; Gavriilaki et al., 2023). Despite its low glycemic index, fructose consumption contributes to insulin resistance, type 2 diabetes, and lipogenesis, which may lead to excessive fat accumulation in blood vessels, thereby elevating the risk of heart disease and persistent hypertension (Martín-Timón, 2014).

Our findings strongly support the association between fructose intake and elevated SBP, leading to hypertension, regardless of dose or duration. Previous research has shown that adult male rats consuming 10–21% fructose beverages experience both weight gain and increased SBP (Toop & Gentili, 2016). Consistent with these observations, our subgroup analysis confirmed the hypertensive effects of fructose across different concentrations and exposure periods.

SBP increased by 28.50 mmHg in rats given 10–30% w/v fructose and by 33.80 mmHg in those receiving 60–75% w/v fructose. While the slightly higher elevation in the higher concentration group suggests a possible dose-dependent effect, but the test for subgroup differences found no significant variation between the two groups, indicating that fructose’s effect on SBP is not influenced by dosage. Furthermore, SBP increased by 31.30 mmHg (*p* < 0.0007) in studies lasting less than 8 weeks, 33.78 mmHg (*P* < 0.00001) in 8-week studies, and 23.95 mmHg (*P* < 0.00001) in studies exceeding 8 weeks. While prolonged fructose exposure may slightly reduce the magnitude of SBP elevation, the consistent and significant increase across all durations highlights fructose’s undeniable role in hypertension development. These findings emphasize the need for further investigation into its long-term impact on cardiovascular health.

### Strain-specific effects on fructose-induced hypertension

One factor that may contribute to variability in blood pressure responses is the strain of rats used in experiments (Kwitek, 2019). Most studies in this meta-analysis involved Wistar and Sprague Dawley (SD) rats, which differ in genetic background, metabolic rate, and susceptibility to diet-induced hypertension (Oron-Herman et al., 2008; Sadie-Van Gijsen & Kotzé-Hörstmann, 2023). While some reports suggest that SD rats may be more resistant to metabolic disturbances than Wistar rats, others indicate no major differences in fructose-induced hypertension between the two strains (Dupas et al., 2017; Cuervo Sánchez et al., 2024). A strain-specific analysis was not conducted in the current meta-analysis, as our primary focus was on evaluating the effects of fructose dose and duration on SBP. While the data includes studies involving different rat strains, such as Sprague Dawley and Wistar rats, our initial research objectives did not prioritize strain-specific comparisons.

Future research should systematically assess strain-dependent variations in blood pressure regulation, metabolic responses, and fructose metabolism efficiency (Bhat et al., 2023). This could provide a more nuanced understanding of the hypertensive effects of fructose and help researchers design more standardized experimental models with greater translational relevance to human health.

### Public health implications

The global rise in processed and canned food consumption has significantly contributed to increased dietary fructose intake, particularly through high-fructose corn syrup (Malik & Hu, 2015). Prior studies reported that fructose beverages at 10–20% w/v concentrations elevate SBP in rodents (Toop & Gentili, 2016), and our meta-analysis further supports this trend in both Wistar and SD rats. Fructose’s adverse cardiovascular effects including abnormal heart, blood vessel functions, and undesirable health outcomes are evident across multiple preclinical models. For instance, studies have demonstrated that dogs consuming 60% of their daily caloric intake from fructose over 20–28 days experience a significant rise in mean arterial pressure (Martinez, Rizza & Romero, 1994). Similarly, a meta-analysis on acute fructose exposure in humans also revealed a significant association with increased SBP, highlighting the immediate and long-term risks of fructose consumption (Siddiqui & Rossi, 2024).

Our study highlights that fructose consumption, whether in high or low concentrations, significantly contributes to an increased risk of hypertension by raising SBP. These findings underscore the need to limit fructose intake, especially from artificially sweetened beverages and processed foods. Public health initiatives should promote healthier alternatives, such as water or non-nutritive sweetened beverages, to help reduce hypertension risk and prevent associated cardiovascular complications.

## Limitations of the Study

This study is limited to animal models, which may restrict the direct applicability of findings to humans due to physiological differences. Additionally, the exact amount of fructose consumed by the animals was not accounted for, meaning that blood pressure changes may be influenced not only by fructose concentration and duration but also by total fructose intake per day. Further studies are needed to establish a clearer link between dietary fructose intake and hypertension risk in both animal and human models.

Despite our efforts to include all relevant studies, asymmetry in the funnel plot suggests potential publication bias, likely due to the underrepresentation of studies with negative or non-significant results. Future research should minimize this bias by incorporating unpublished data including preprints, grey literature, and direct researcher contributions where possible and expanding searches to include gray literature for a more comprehensive analysis.

## Conclusion

Fructose may influence blood pressure in animals by affecting various aspects of the cardiovascular system. This meta-analysis examined the impact of fructose dosage and duration on SBP. The findings suggest that prolonged exposure to physiological concentrations of fructose (10% w/v for more than 8 weeks) or short-term administration of supraphysiological concentrations (60% w/v) tends to elevate blood pressure, although not all findings reached statistical significance. These results highlight the potential risk associated with excessive fructose intake, particularly in hypertensive conditions. Reducing dietary intake of added sugars, including artificial sweeteners, remains a prudent strategy to mitigate possible cardiovascular risks.

## Supplemental Information

10.7717/peerj.20097/supp-1 Supplemental Information 1 PRISMA checklist

## Additional Information and Declarations

### Data Availability

The following information was supplied regarding data availability: This is a systematic review/meta-analysis.
